# Evaluating scenarios for school reopening under COVID19

**DOI:** 10.1186/s12889-022-12910-w

**Published:** 2022-03-14

**Authors:** Arden Baxter, Buse Eylul Oruc, John Asplund, Pinar Keskinocak, Nicoleta Serban

**Affiliations:** 1 H. Milton Stewart School of Industrial and Systems Engineering, Georgia Institute of Technology, Atlanta, GA USA; 2 Metron, Inc., Reston, VA USA; 3 Rollins School of Public Health, Emory University, Atlanta, GA USA

**Keywords:** COVID19, Pandemic, Public health, School reopening, Non-pharmaceutical interventions, Agent-based disease modeling

## Abstract

**Background:**

Thousands of school systems have struggled with the decisions about how to deliver education safely and effectively amid the COVID19 pandemic. This study evaluates the public health impact of various school reopening scenarios (when, and how to return to in-person instruction) on the spread of COVID19.

**Methods:**

An agent-based simulation model was adapted and used to project the impact of various school reopening strategies on the number of infections, hospitalizations, and deaths in the state of Georgia during the study period, i.e., February 18^th^-November 24^th^, 2020. The tested strategies include (i) *schools closed*, i.e., all students receive online instruction, (ii) *alternating school day*, i.e., half of the students receive in-person instruction on Mondays and Wednesdays and the other half on Tuesdays and Thursdays, (iii) *alternating school day for children*, i.e., half of the children (ages 0-9) receive in-person instruction on Mondays and Wednesdays and the other half on Tuesdays and Thursdays, (iv) *children only*, i.e., only children receive in-person instruction, (v) *regular*, i.e., all students return to in-person instruction. We also tested the impact of universal masking in schools.

**Results:**

Across all scenarios, the number of COVID19-related deaths ranged from approximately 8.8 to 9.9 thousand, the number of cumulative infections ranged from 1.76 to 1.96 million for adults and 625 to 771 thousand for children and youth, and the number of COVID19-related hospitalizations ranged from approximately 71 to 80 thousand during the study period. Compared to schools reopening August 10 with a *regular* reopening strategy, the percentage of the population infected reduced by 13%, 11%, 9%, and 6% in the *schools closed*, *alternating school day for children*, *children only*, and *alternating school day* reopening strategies, respectively. Universal masking in schools for all students further reduced outcome measures.

**Conclusions:**

Reopening schools following a *regular* reopening strategy would lead to higher deaths, hospitalizations, and infections. Hybrid in-person and online reopening strategies, especially if offered as an option to families and teachers who prefer to opt-in, provide a good balance in reducing the infection spread compared to the *regular* reopening strategy, while ensuring access to in-person education.

**Supplementary Information:**

The online version contains supplementary material available at 10.1186/s12889-022-12910-w.

## Background

School systems have developed plans for safely reopening during the fall semester of 2020 while considering the potential impact of in-person interactions on students, staff, families, and public health during the COVID19 pandemic [1–3]. Studies have shown the potential benefits of non-pharmaceutical interventions, such as school and workplace closures [4–7], in slowing down infection spread and reducing the severe health outcomes, but also highlighted their negative impact on the economy, unemployment, mobility, mental health, education, caregiving, etc. [8–10]. Widespread school closures during spring 2020 not only impacted the education of students but also had social and economic consequences, e.g., due to increased childcare responsibilities of working parents [11–19]. In addition, increased childcare responsibilities may also lead to worker absenteeism in healthcare and further exacerbate the already stressed healthcare system [20].

Agent-based models capture complex interactions and processes, such as interventions, adaptive behaviors, and contextual effects across subpopulations [21]. They are particularly helpful in forecasting the spread of emerging infectious diseases by coalescing disease dynamics, pharmaceutical and non-pharmaceutical interventions, and social behaviors, potentially leading to a better understanding of the disease spread and corresponding actions [22]. In this study, we adapted an agent-based simulation model to predict public health outcomes (e.g., number of community-wide infections, hospitalizations, and deaths) under various school reopening scenarios. School reopening scenarios considered different in-person versus online participation options and interventions, such as symptom-based self-isolation and universal masking in schools.

We considered school reopening scenarios for the fall semester of 2020 in the state of Georgia. At this time, COVID19 infections were on the rise due to social unrest and increased mobility [23–27]. Further, vaccines were not widely available [28] and data on levels of compliance with social distancing measures was unreliable [29, 30]. While we use fall 2020 as a template for our study, the methods and insights are applicable to future pandemic situations when cases are on the rise and information is limited. The insights of our study would also be helpful if a major variant of the SARS-CoV-2 virus emerges and significantly reduces the effectiveness of existing vaccines.

The objective of this study is to quantify the public health outcomes (deaths, hospitalizations, and infections) of various school reopening scenarios to measure their impact on the spread of a pandemic in an effort to provide much-needed insights for school system decision-makers amid an ongoing pandemic.

## Methods

### Model description

The results were obtained by adapting and utilizing an agent-based simulation model to predict the spread of COVID19 geographically and over time [31–34]. An agent-based simulation model is a computational model that simulates a number of autonomous “agents”, where each agent represents an individual in the population, and the model mimics the dynamics and outcomes of a real system under certain assumptions. The study population represented in the model consisted of children (ages 0-9), youth (ages 10-19), adults (ages 20-64), and elderly (ages 65+), mimicking the population characteristics in the state of Georgia. The state of Georgia has a total population of approximately 10.8 million where 1.3 million are children and 1.4 million are youth [35]. The simulation consisted of one million agents (i.e., approximately one agent per ten individuals with similar characteristics in the population), to capture the population dynamics while keeping the run times reasonable. The model captured the progression of the disease in an individual and interactions within households, workplaces, schools, and communities. All children and youth ages 0-19 in Georgia were assumed to be in a peer group of similar age (e.g., school, daycare, etc.). School-based peer interactions occur between individuals within their assigned age groups. Peer groups were chosen on average as 14, 20, and 30 students for age groups 0-4, 5-9, and 10-19, respectively, following average class sizes reported in Georgia [36]. Interactions between different age groups occur within household and community settings. The model enabled the testing of scenarios incorporating various types and durations of physical distancing interventions, namely school closures, shelter-in-place, voluntary quarantine of households (i.e., the entire household remains home if there is a person in the household with cold/flu-like symptoms), and masking. These interventions are defined as follows:

- *Shelter-in-place:* Staying home and refraining from interactions outside of the household.
- *Voluntary quarantine:* Members of a household stay home if there is a person in the household with cold/flu-like symptoms, until the entire household is symptom-free.
- *Voluntary shelter-in-place:* An entire household chooses to remain home, even if there is no shelter-in-place order, and regardless of symptoms.
- *Voluntary masking:* Wearing a face covering/mask while interacting outside of the household.

Additionally, we considered the following school-based interventions:

- *Symptom-based self-isolation:* Students who present with COVID19 symptoms do not engage in school-based peer interactions.
- *Universal masking in schools:* All students are required to wear a face covering/mask while attending in-person instruction. Masks are assumed to reduce the infectivity and susceptibility of individuals by 50%.

The model captures the public’s varying level of compliance with these interventions [37–39] using the demographic and workflow information at the census tract level in Georgia. The initial infections “seeds” follow the distribution of the total number of confirmed COVID19 cases in Georgia (as of May 15, 2020) [29, 40]. Additional information about the model and the data sources can be found in Supplementary Section A and Supplementary Fig. 1, Additional File 1, and in [31, 32]. The study period is from February 18 to November 24, 2020.

## School reopening strategies

- *Schools closed:* All students receive online instruction.
- *Alternating school day:* Half of the students receive in-person instruction on Mondays and Wednesdays and the other half on Tuesdays and Thursdays.
- *Alternating school day for children:* Half of the children (ages 0-9) receive in-person instruction on Mondays and Wednesdays and the other half on Tuesdays and Thursdays.
- *Children only:* Only children receive in-person instruction.
- *Regular:* All students return to in-person instruction.

**Fig. 1:**
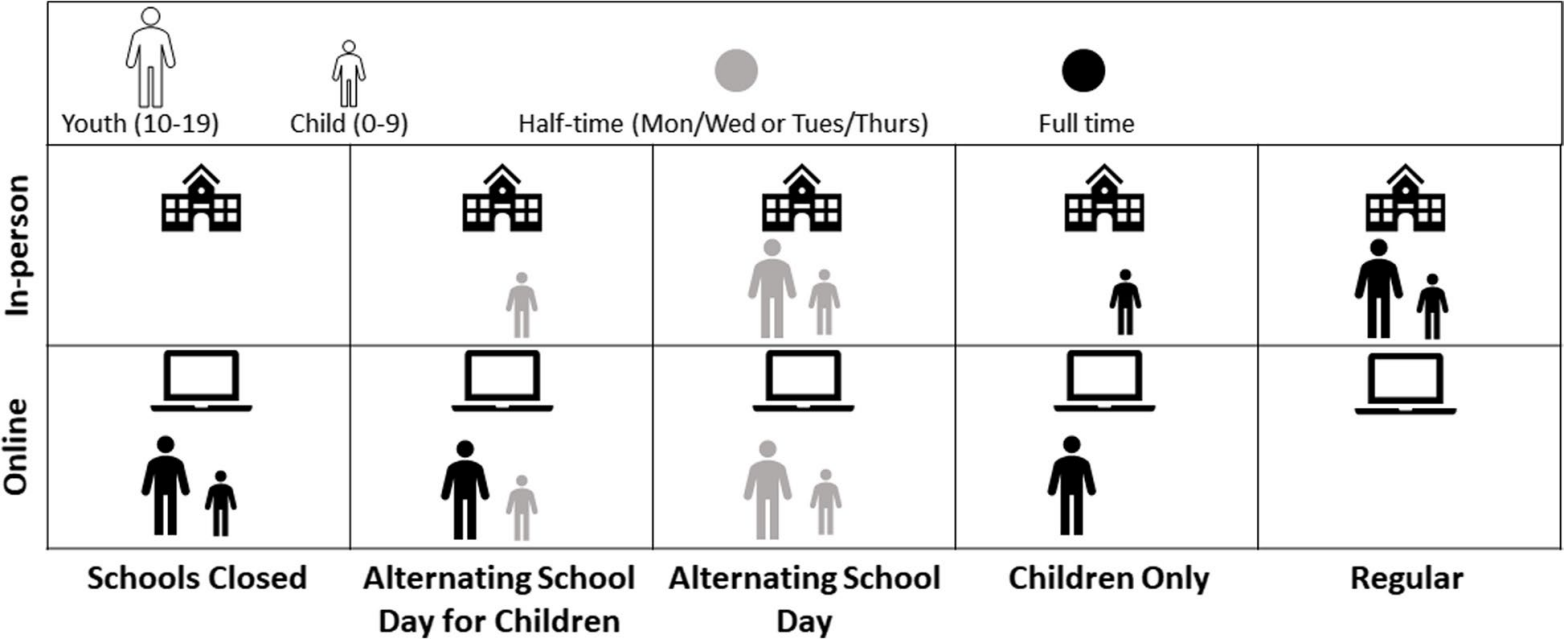
Description of school reopening strategies

On each day, the school status (“attending in-person" or “attending online”) of children (0-9) and youth (10-19) was tracked and updated in the simulation depending on the chosen school reopening strategy (see Fig. 1 for the description of school reopening strategies). Students “attending online” did not engage in school-based peer interactions and are assumed to stay at home while still interacting in the community.

## Scenario descriptions

**Fig. 2:**
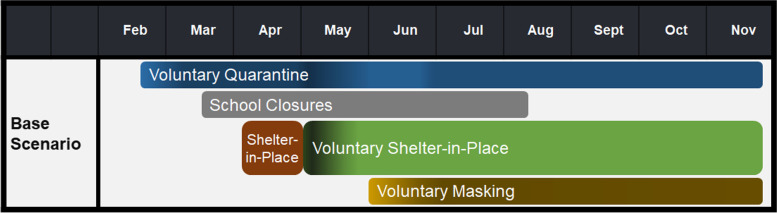
Base Scenario. All scenarios considered are built upon the base scenario along with the corresponding school reopening date. Compliance with shelter-in-place, voluntary quarantine, and voluntary shelter-in-place varies over time

To model the spread of COVID19 in the state of Georgia prior to the start of the fall semester of 2020, the simulation incorporated school closures during March 16- August 10 [41] and physical distancing practices for the entire population with varying levels of compliance (see Fig. 2). In Georgia, the shelter-in-place order was in place during April 3-30, 2020. Shelter-in-place compliance of 80% was assumed for that time period [31]. Voluntary quarantine compliance was 30% in mid-February, increased by 10% weekly until mid-March, and remained at 60% until the end of April. After the end of shelter-in-place, voluntary quarantine compliance was 70% and decreased by 10% weekly until stabilizing at 50% with 40% during the weeks of social unrest (from May 29^th^ to June 23^rd^, 2020) [23–26]. During the week after the end of shelter-in-place, voluntary shelter-in-place compliance was 60% and decreased to 40%, 20%, and 5%, in consecutive weeks, until stabilizing at 5%. We assume that masks reduce the infectivity and susceptibility of individuals by 50% [42]. In the first two weeks of June, mask compliance in peer groups and the community is assumed to be 20%, later increasing 10% weekly until reaching 60% compliance [43, 44]. Voluntary shelter-in-place and voluntary quarantine compliance levels in the model were chosen to be in line with social mobility indicators [27]. A validation of our model results can be found in Supplementary Section B and Supplementary Fig. 2, Additional File 1.

The first set of scenarios considered in this study were each school reopening strategy with different reopening dates. Kindergarten through 12^th^ grade (K-12) schools typically open during the first or second week of August in Georgia; hence, the following reopening dates were considered; August 10, August 17, August 24, August 31, September 7, and September 14 (represented with numbers 1 through 6, respectively, in scenario labeling). In the tables and figures that follow, these scenarios were labeled by their names, as well as numbers 1 through 6. For example, *Alternating School Day 3* refers to the scenario in which schools are reopened on August 24 and students adhere to an *alternating school day* reopening strategy.

In the next set of scenarios, we fixed the reopening date to August 10 and considered each school reopening strategy, along with universal masking in schools. In the tables and figures that follow, these scenarios are labeled by their names, followed by *M* to denote the inclusion of universal masking in schools (e.g., *Children Only M*).

All scenarios tested were built upon the base scenario described in Fig. 2 and students adhere to symptom-based self-isolation.

### Outcome measures

The infection spread outcome measures during the study period included:

- *New daily infections:* Number of people infected with COVID19 on a given day.
- *Cumulative infections:* Cumulative number of people infected (including asymptomatic infections).
- *Infection attack rate (IAR):* Cumulative percentage of the population infected.
- *Peak day:* The day when the number of new infections was highest.
- *Peak infection:* The number of the population infected on the peak day.
- *Cumulative deaths:* Cumulative number of people who died due to COVID19.
- *Cumulative hospitalizations:* Cumulative number of people hospitalized due to COVID19.

## Results

Depending on the school reopening date and strategy, school-based and other interventions, and the public’s compliance with physical distancing, the number of COVID19-related deaths during the study period could range from approximately 8.8 to 9.9 thousand in the state of Georgia and the number of cumulative infections could range from 1.76 to 1.96 million for adults and 625 to 771 thousand for children and youth. The number of COVID19-related hospitalizations could range from approximately 71 to 80 thousand. Compared to all other scenarios, under the *regular* reopening strategy with a reopening date of August 10, on average, the peak infection in children and youth is 21% higher and the peak day is August 22 (almost one month later than that of other scenarios). Tables 1 and 2 provide results for outcome measures under all scenarios considered.

**Table 1:** Outcome measures across scenarios without universal masking in schools

**Reopening Date**	**Reopening Strategy**	**Cumulative Deaths**	**Cumulative Hosp.**	**Cumulative Infections**		**IAR %**	**Peak Infections**		**Peak Day**
				**Children & Youth**	**Adult**		**Children & Youth**	**Adult**	
-	Schools Closed	8894	71658	625238	1757331	22.06	4685	13553	21-Jul
1	Alternating School Day for Children	9032	73148	646753	1794362	22.61	4785	13699	24-Jul
2		9147	73281	643065	1789006	22.52	4682	13778	20-Jul
3		8976	72799	640933	1782047	22.44	4763	13725	27-Jul
4		8884	72243	636193	1773745	22.32	4633	13436	28-Jul
5		8983	72780	636659	1780587	22.39	4764	13728	23-Jul
6		9146	73085	638953	1787263	22.47	4775	13669	24-Jul
1	Alternating School Day	9458	76563	699242	1870620	23.80	4834	13861	24-Jul
2		9381	75569	685670	1850710	23.49	4731	13742	24-Jul
3		9255	75041	676364	1831377	23.22	4642	13627	26-Jul
4		9154	74134	667963	1818019	23.02	4681	13526	22-Jul
5		9211	74077	664232	1814629	22.96	4819	13773	24-Jul
6		9149	73483	656232	1800472	22.75	4650	13606	23-Jul
1	Children Only	9272	74227	668797	1818029	23.03	4718	13634	24-Jul
2		9226	74282	663668	1814065	22.95	4789	13726	24-Jul
3		9068	73680	656247	1797326	22.72	4749	13684	24-Jul
4		9195	73555	656817	1806753	22.81	4755	13819	24-Jul
5		8911	72094	639715	1762858	22.25	4587	13405	23-Jul
6		9149	73270	645458	1791968	22.57	4691	13814	21-Jul
1	Regular	9744	80263	778272	1962672	25.38	5721	12728	22-Aug
2		9799	79422	760373	1941517	25.02	4677	13731	26-Jul
3		9642	78414	745511	1924197	24.72	4713	13683	23-Jul
4		9531	77770	730702	1903714	24.40	4748	13994	22-Jul
5		9351	76124	711876	1871483	23.92	4838	13948	24-Jul
6		9296	74954	691499	1837665	23.42	4698	13704	23-Jul

Summary comparison of school reopening scenarios without universal masking in schools with respect to cumulative deaths and hospitalizations in the total population, cumulative infections in adults and children/youth, infection attack rate (IAR) in the total population, peak infections in adults and children/youth, and peak day in the total population. Numbers 1-6 correspond to dates Aug 10, Aug 17, Aug 24, Aug 31, Sept 7, and Sept 14, respectively

**Table 2:** Outcome measures across scenarios with universal masking in schools

**Reopening Strategy**	**Cumulative Deaths**	**Cumulative Hosp.**	**Cumulative Infections**		**IAR %**	**Peak Infections**		**Peak Day**
			**Children & Youth**	**Adult**		**Children & Youth**	**Adult**	
Schools Closed	8894	71658	625238	1757331	22.06	4685	13553	21-Jul
Alternating School Day for Children	9113	73329	645689	1793551	22.59	4854	14018	26-Jul
Alternating School Day	9390	75805	689727	1855026	23.57	4728	13805	27-Jul
Children Only	9240	73847	669356	1811767	22.98	4687	13679	24-Jul
Regular	9883	79734	771052	1950509	25.20	4660	13463	27-Jul

Summary comparison of school reopening strategies with universal masking during in-person education with respect to cumulative deaths and hospitalizations in the total population, cumulative infections in adults and children/youth, infection attack rate (IAR) in the total population, peak infections in adults and children/youth, and peak day in the total population

### Impact of school reopening date

**Fig. 3:**
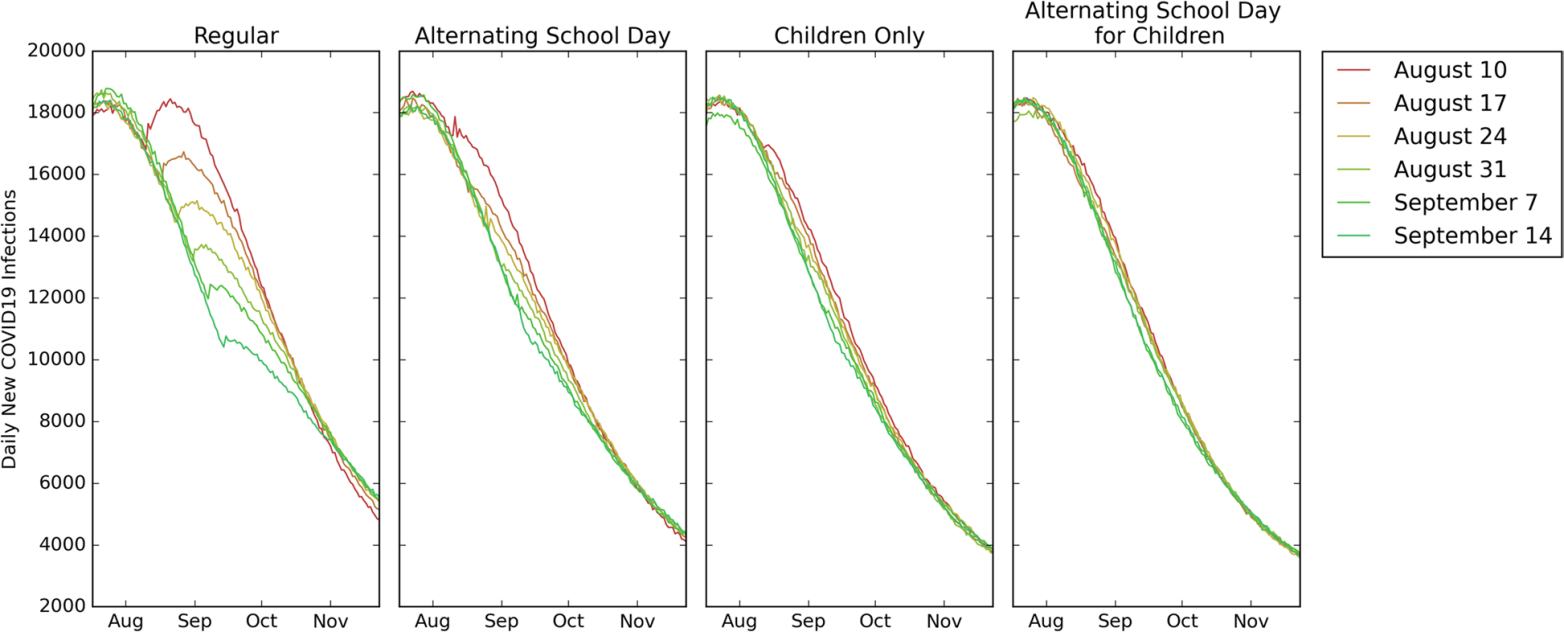
Impact of reopening date given a reopening strategy. Daily new COVID19 infections with different reopening dates under the *regular, children only*, *alternating school day*, and *alternating school day for children* reopening strategies

Delaying the school reopening date provided the greatest benefit when implementing a *regular* reopening strategy. Under the *regular* reopening strategy, delaying the reopening date by one week reduced the cumulative infections in children and youth by at most 3% and reduced all other outcome measures (cumulative deaths, hospitalizations, and cumulative infections in adults) by at most 2%. Delaying the school reopening date by 5 weeks reduced the cumulative deaths by 5%, cumulative hospitalizations by 7%, cumulative infections in children and youth by 11%, and cumulative infections in adults by 6%. In all other reopening strategies considered, delaying the school reopening date didn’t provide as much benefit compared to the *regular* reopening strategy. On average, across all school reopening strategies, delaying the reopening date by one week provided a 0.39% reduction in cumulative deaths, a 0.61% reduction in hospitalizations, a 1.13% reduction in cumulative infections in children and youth, and a 0.61% reduction in cumulative infections in adults. Fig. 3 presents a comparison of daily new infections under different reopening dates for a given school reopening strategy.

### Impact of school reopening strategies

Compared to schools reopening on August 10 with a *regular* reopening strategy, the percentage of the population infected reduced by 13%, 11%, 9%, and 6% in the *schools closed, alternating school day for children, children only,* and *alternating school day* reopening strategies, respectively.

For the reopening date of August 10, compared to the *schools closed* reopening strategy*:*

- Deaths increased by 138, 379, 564, and 851 in the *alternating school day for children only, children only, alternating school day, and regular* reopening strategies, respectively*.*
- Hospitalizations increased by 1,490; 2,569; 4,905; and 8,605 in the *alternating school day for children only*, *children only, alternating school day*, and *regular* reopening strategies, respectively.
- Cumulative infections in children and youth increased by 21,514; 43,558; 74,000; and 153,033 in the *alternating school day for children only*, *children only*, *alternating school day*, and *regular* reopening strategies, respectively.
- Cumulative infections in adults increased by 37,031; 60,698; 113,289; and 205,341 in the *alternating school day for children only, children only, alternating school day, and regular* reopening strategies, respectively.

**Fig. 4:**
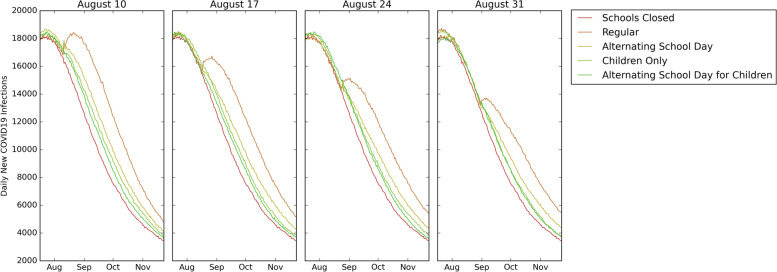
Impact of reopening strategy given a reopening date. Daily new COVID19 infections with different reopening strategies under the reopening dates of Aug 10, Aug 17, Aug 24, Aug 31

Results for other reopening dates are similar to those presented above. Fig. 4 shows a comparison of the daily new infections under different school reopening strategies while fixing the reopening dates to August 10, August 17, August 24, and August 31.

### Impact of universal masking in schools

**Fig. 5:**
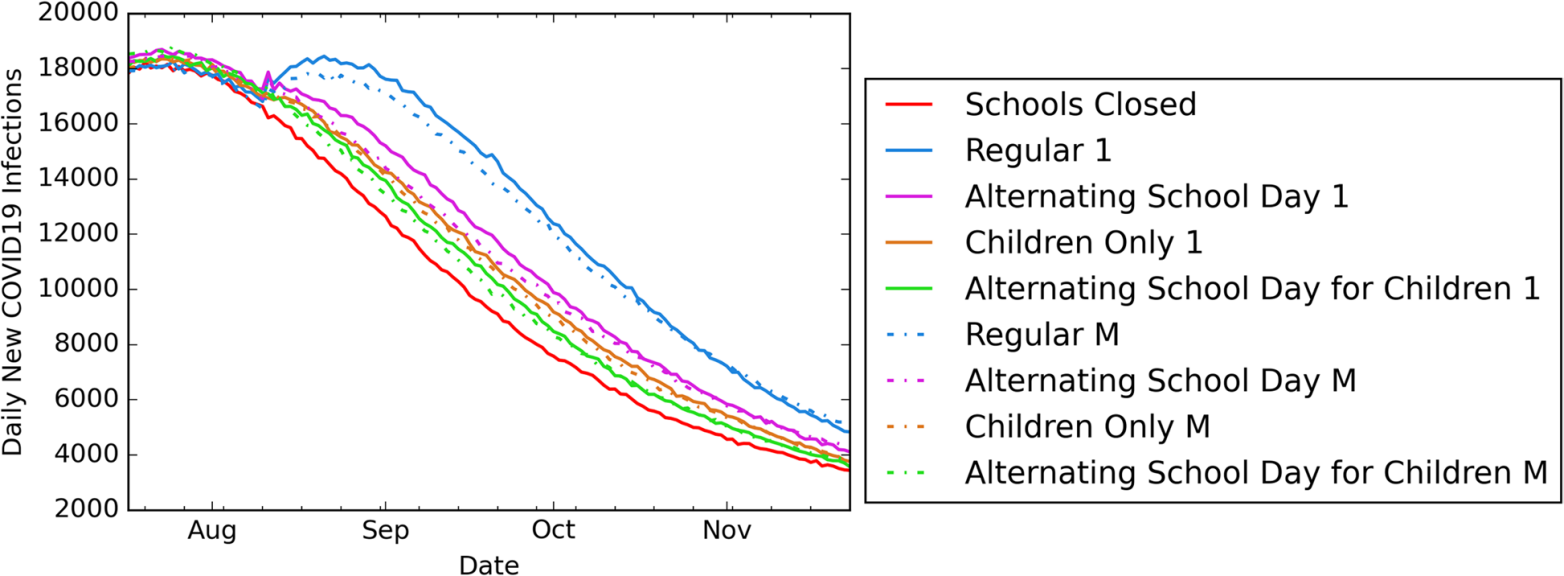
Impact of universal masking in school. Daily new COVID19 infections with different reopening strategies with or without universal masking in schools under the reopening date of Aug 10

Figure 5 presents a comparison of the daily new infections under different school reopening strategies with and without universal masking in schools. For the reopening date of August 10, universal masking in schools decreased deaths by at most 0.72%, hospitalizations by at most 0.99%, cumulative infections in children and youth by at most 1.36%, and cumulative infections in adults by at most 0.83%. On average, universal masking in schools reduced the cumulative infections in children and youth and in adults by 0.59% and 0.46%, respectively. The addition of universal masking in schools to school reopening strategies reduced outcome measures; however, compared to the *schools closed* reopening strategy, deaths, hospitalizations, and cumulative infections were higher by 2-3%, 3-7%, 6-10%, and 11-23% in the *alternating school day for children, children only*, *alternating school day*, and *regular* school reopening strategies, respectively.

## Discussion

Governments and school systems have grappled with the decisions of how to prepare students for academic success while also trying to minimize the spread of COVID19. While individuals younger than 20 seemed to be less affected by COVID19 than adults, they could be transmitters of COVID19, potentially increasing community infection spread if schools were to return to in-person instruction [6, 19, 45], particularly considering the challenges in the implementation of social distancing measures and recommendations for some schools (e.g., poor ventilation in buildings, short supply of disinfectant products, state budget shortfalls, etc.) [1, 10].

The negative impact of school closures has been disproportionately high on some students, e.g., those who do not have access to technology in the household, lack proper childcare, face an unsafe home environment, or have traditionally relied on the school system for meals, special education, counseling, and other forms of social or emotional support [10, 46]. School closures could lead to numerous unintended negative consequences as well, such as increased worker absenteeism among parents; higher worker absenteeism within the healthcare system could increase case-fatality risk and the overall mortality rate due to the pandemic [12].

Prior to the start of the fall semester in 2020, guidelines in the state of Georgia recommended for districts with high case numbers to reopen schools with online instruction. However, online instruction poses numerous challenges. Several rural counties have limited internet connectivity; for example, Hancock County ranked number six for COVID19 cases per capita yet only 2% of the county has access to broadband internet [47]. Two-thirds of students in Georgia are not able to read proficiently by the end of third grade; limited or no access to in-person instruction in the fall could further increase this educational gap, with significant long-term consequences [10, 48]. Further, in Georgia, over half of the students are eligible for free and reduced fee school lunches and many families depend on these services [49].

There has been considerable debate about the benefits and risks of when and how to return to in-person instruction in schools during fall 2020. The American Academy of Pediatrics “strongly advocates that all policy considerations for the coming school year should start with a goal of having students physically present in school [50].” Some school systems delayed their opening dates or announced fully online instruction for the fall semester, while others considered hybrid models such as “groups of students to attend on alternating days or weeks, as well as allowing only younger students to attend while older students learn at home [51].”

This study compared the impact of various school reopening strategies, in the presence of a combination of other non-pharmaceutical interventions such as shelter-in-place, voluntary quarantine of households, and masking, with varying levels of compliance, along with universal masking and symptom-based isolation in schools. According to our study results, delaying the reopening date would have a minimal impact on the peak day and peak number of new infections under the *alternating school day*, the *children only*, and the *alternating school day for children* reopening strategies. However, under the *regular* reopening strategy, delaying the reopening date from August 10 to September 17 could avoid the second peak and reduce the peak number of new children and youth infections by 22%.

Compared to the *schools closed* reopening strategy, the *alternating school day for children* reopening strategy increased deaths, hospitalizations, and cumulative infections the least, followed by the *children only* and then the *alternating school day* reopening strategies. Hybrid reopening strategies such as the *alternating school day for children*, *children only*, and *alternating school day* significantly reduced the percentage of the population infected when compared to the *regular* reopening strategy (by 6-13%). Hence, implementing a hybrid reopening strategy or limiting interactions between student cohorts during the in-person instruction could have a significant impact on slowing down the disease spread. A population study focused on Washington and Michigan found that overall, online instruction or providing hybrid instruction led to fewer infections compared to in-person instruction, supporting the findings in this study [52]. The addition of universal masking to in-person instruction further reduced infections, hospitalizations, and deaths.

## Conclusions

COVID19 has had a significant impact on society both in terms of public health and social and economic interactions. The health and well-being of the population are of the utmost importance, but there is also a growing desire to return to in-person instruction to support the educational development of students.

As school systems develop plans for modes of instruction during an epidemic or pandemic, it is critical to understand the impact of various scenarios on public health as well as students’ development and the economy. Our results suggest that opening schools following a *regular* reopening strategy, i.e., all students returning to school without strict public health measures, would significantly increase the number of infections, hospitalizations, and deaths. Hybrid in-person and online reopening strategies, especially if offered as an option to families and teachers who prefer to opt-in, provide a good balance in reducing the infection spread compared to the *regular* reopening strategy*,* while ensuring access to in-person education.

The impact of other non-pharmaceutical public health measures, including workplace closings, voluntary quarantine compliance, shelter-in-place, and masking mandates have been considered and compared in prior work [7, 9, 20, 31, 53–56]. Some studies found that the public health effects of school closures are similar to that of workplace closures [20, 53, 54]. Our study focused on the impact of various school reopening scenarios to provide policy suggestions to decision-makers amid an ongoing pandemic or epidemic, in the presence of other non-pharmaceutical interventions. Regardless of how school instruction is formatted during a pandemic, it is important to promote physical distancing measures, vaccination, and the usage of face masks as well as establishing testing and tracing practices to ensure prevention or early detection of outbreaks in schools.

## Supplementary Information

**Additional file 1. **

## Data Availability

The datasets generated during and/or analyzed during the current study are available from the corresponding author on reasonable request. All data used was publicly available.

## Abbreviations

M: Universal masking in schools
K-12: Kindergarten through 12^th^ grade
IAR: Infection Attack Rate
