# Supplementary material for: Evaluating scenarios for school reopening under COVID19
Source: BMC Public Health. 2022 Mar 14;22:496. doi: 10.1186/s12889-022-12910-w (PMC8919143; doi:10.1186/s12889-022-12910-w)
Supplement: Supplementary file 1 — Additional file 1. [file 12889_2022_12910_MOESM1_ESM.docx]

**Evaluating Scenarios for School Reopening under COVID19 Supplementary Material**

**Supplementary Section A: Model Details**

We adapted and utilized an agent-based simulation model that tracks both the natural progression of disease in each individual and the overall spread of the disease. In the simulation model, there is an “agent” representing an individual in the population. To realistically capture the population dynamics while keeping the simulation run time reasonable, there are 1 million agents in the simulation representing the population of 10.8 million in the state of Georgia.

Individual disease progression is modeled by a modified Susceptible-Exposed-Infected-Recovered (SEIR) model (see Supplementary Figure 1), where each individual is in one of the following states at any particular time in the simulation: susceptible (S), exposed (E), transitioning (from exposed to asymptomatic or symptomatic) (IP), asymptomatic (IA), symptomatic (IS), hospitalized (H), recovered (R), or dead (D). The time spent in any of these states follows a probability distribution, as stated in Supplementary Table 1. The probability of transitioning to a more severe disease state, such as hospitalized or dead, varies by age group. Each agent in the simulation has certain attributes and belongs to a household, a community, and a peer group (e.g., workplace for adults and schools for children and youth), which are populated with data (e.g., demographic data, household statistics, classroom sizes) from the state of Georgia [1]. For example, the household size and the age distribution of household members varies across geographic areas of the state. Contact between individuals (agents) in the simulation occurs through interactions in households, schools, workplaces, and communities; population mixing occurs in households at night, peer groups during the day, and in communities both day and night (see Supplementary Figure 1). The simulation model also captures work-related travel between different areas of the state, utilizing workflow data [2]. The agent-based simulation model was implemented using C++. For each scenario, the model is run 30 times, and the results presented are the averages of these 30 runs.

Further details on the data sources, model description, and model parameters, as well as simulation validation can be found in [3, 4].

**Supplementary Figure 1. Model description.** Description of the modified SEIR model used to track individual disease progression and contacts between individuals modeled in the agent-based simulation.


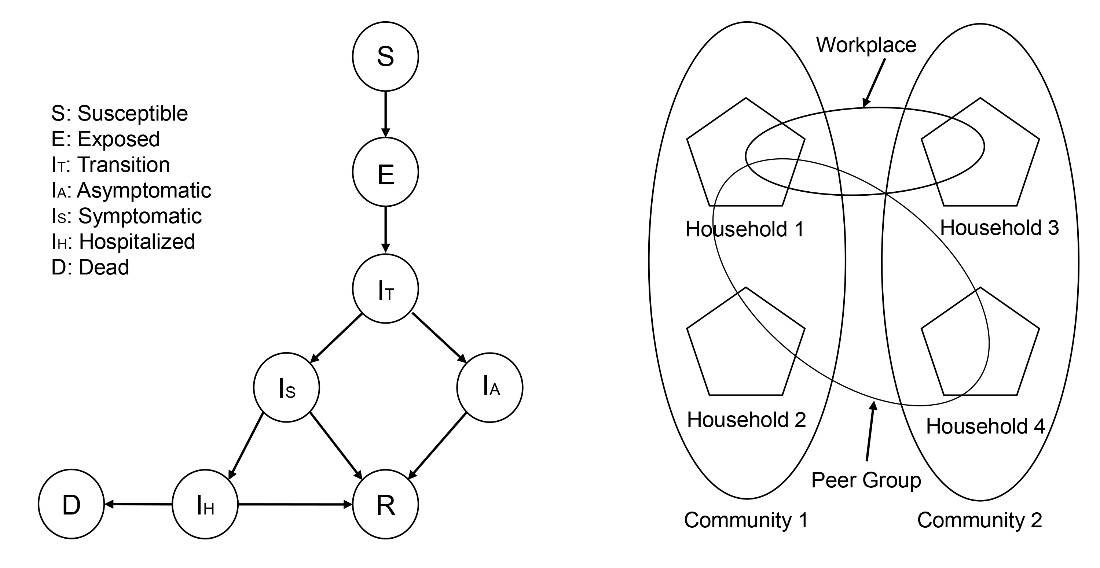


**Supplementary Table 1. Model parameters.** Description of model parameters, values and references.

| **Parameters** | **Estimates** | **References** |
| --- | --- | --- |
| Probability of Symptomatic | 0.63 | [5] |
| Probability of Hospitalization | 0.016 for age 0-19,  0.18 for age 20-64,  0.30 for age 65+ | [6] |
| Probability of Death | 0 for age 0-19,  0.103 for age 20-64,  0.375 for age 65+ | [5, 6] |
| Reproductive Number ($R_{0}$) | 2.4 | [7-9] |
| Transmission Rate (*β*) | 1.02 | [8] |
| Exposed Duration | Weibull with mean 4.6 days | [10-13] |
| Transition Duration | 0.5 days | [11] |
| Hospitalized Duration | Exponential with mean 7 days | [5, 11, 14] |
| Symptomatic Duration | Exponential with mean 2.9 days | [15] |
| Symptomatic-Asymptomatic Duration Ratio | 1.5 | [11] |

**Supplementary Section B: Model Validation**

Supplementary Figure 2 presents a validation of our model results by comparing the simulation infection and death predictions with the estimated actual infections and deaths in Georgia. The confirmed number of daily new COVID19 infections in Georgia was multiplied by a factor of 4-8 to account for under-reporting, under-testing, and the existence of asymptomatic infections [16, 17]. The confirmed number of deaths in Georgia was multiplied by 1-1.25 to account for under-counting [18].

**Supplementary Figure 2. Model validation.** Daily new COVID19 infections (left plot) and cumulative number of COVID19 deaths (right plot) under *schools closed* reopening strategy considering behavioral shifts compared to confirmed numbers of Georgia.


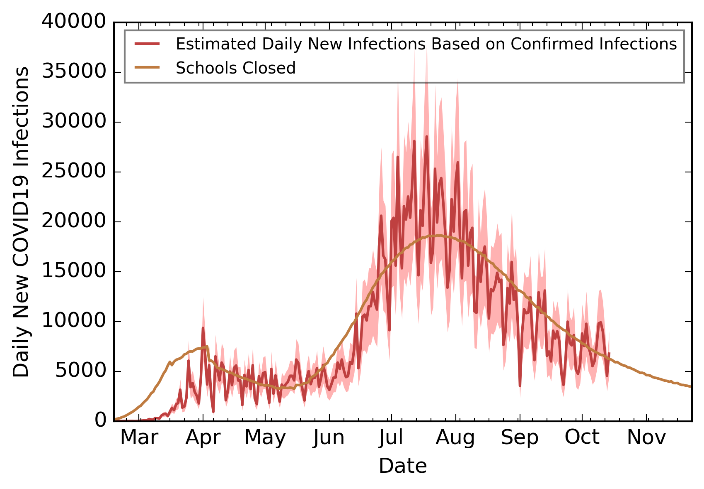

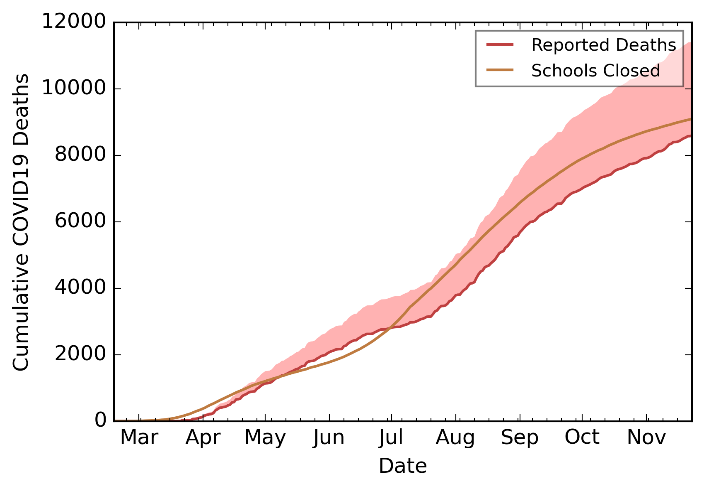


**References**

1. U.S. Census Bureau: **American Community Survey, 2018 American Community Survey 1-year Estimates**. In*.*: U.S. Census Bureau; 2018.

2. U.S. Census Bureau: **Census Transportation Planning Products, 5-year data**. In*.*: U.S. Census Bureau; 2016.

3. Keskinocak P, Oruc BE, Baxter A, Asplund J, Serban N: **The impact of social distancing on COVID19 spread: State of Georgia case study**. *PLoS One* 2020, **15**(10):e0239798.

4. Oruc BE, Baxter A, Keskinocak P, Asplund J, Serban N: **Homebound by COVID19: The Benefits and Consequences of Non-Pharmaceutical Intervention Strategies**. In*.*; 2020.

5. **COVID-19 Pandemic Planning Scenarios** [<https://www.cdc.gov/coronavirus/2019-ncov/hcp/planning-scenarios.html>]

6. CDC COVID-19 Response Team: **Severe Outcomes Among Patients with Coronavirus Disease 2019 (COVID-19) — United States, February 12–March 16, 2020**. In*.*, vol. 69. Morbidity and Mortality Weekly Report (MMWR): CDC; 2020.

7. Walker PG, Whittaker C, Watson O, Baguelin M, Ainslie KEC, Bhatia S, Bhatt S, Boonyasiri A, Boyd OC, Lorenzo, Cucunubá Z *et al*: **The Global Impact of COVID-19 and Strategies for Mitigation and Suppression**. In*.*: Imperial College COVID-19 Response Team; 2020.

8. Li R, Pei S, Chen B, Song Y, Zhang T, Yang W, Shaman J: **Substantial undocumented infection facilitates the rapid dissemination of novel coronavirus (SARS-CoV2)**. *Science* 2020:eabb3221.

9. WHO: **Report of the WHO-China Joint Mission on Coronavirus Disease 2019 (COVID-19)** In*.*: World Health Organization; 2020.

10. Linton NM, Kobayashi T, Yang Y, Hayashi K, Akhmetzhanov AR, Jung S-m, Yuan B, Kinoshita R, Nishiura H: **Incubation Period and Other Epidemiological Characteristics of 2019 Novel Coronavirus Infections with Right Truncation: A Statistical Analysis of Publicly Available Case Data**. *medRxiv* 2020:2020.2001.2026.20018754.

11. Ferguson NM, Laydon D, Nedjati-Gilani G, Imai N, Ainslie K, Baguelin M, Bhatia S, Boonyasiri A, Cucunubá Z, Cuomo-Dannenburg G *et al*: **Impact of non-pharmaceutical interventions (NPIs) to reduce COVID19 mortality and healthcare demand**. In*.*: Imperial College London; 2020.

12. Backer JA, Klinkenberg D, Wallinga J: **Incubation period of 2019 novel coronavirus (2019-nCoV) infections among travellers from Wuhan, China, 20–28 January 2020**. *Eurosurveillance* 2020, **25**(5):2000062.

13. Mizumoto K, Kagaya K, Zarebski A, Chowell G: **Estimating the asymptomatic proportion of coronavirus disease 2019 (COVID-19) cases on board the Diamond Princess cruise ship, Yokohama, Japan, 2020**. *Eurosurveillance* 2020, **25**(10):2000180.

14. Weitz J: **Intervention Serology and Interaction Substitution: Exploring the Role of `Immune Shielding' in Reducing COVID-19 Epidemic Spread** In*.*; 2020.

15. Riou J, Hauser A, Counotte MJ, Althaus CL: **Adjusted age-specific case fatality ratio during the COVID-19 epidemic in Hubei, China, January and February 2020**. *medRxiv* 2020:2020.2003.2004.20031104.

16. **Georgia Department of Public Health COVID-19 Daily Status Report** [<https://dph.georgia.gov/covid-19-daily-status-report>]

17. **Commercial Laboratory Seroprevalence Survey Data** [<https://www.cdc.gov/coronavirus/2019-ncov/cases-updates/commercial-lab-surveys.html>]

18. Weinberger DM, Chen J, Cohen T, Crawford FW, Mostashari F, Olson D, Pitzer VE, Reich NG, Russi M, Simonsen L *et al*: **Estimation of Excess Deaths Associated With the COVID-19 Pandemic in the United States, March to May 2020**. *JAMA Internal Medicine* 2020.
